# Is Political Activism on Social Media an initiator of Psychological Stress?

**DOI:** 10.12669/pjms.336.12863

**Published:** 2017

**Authors:** Aliya Hisam, Iqra Safoor, Nawal Khurshid, Aakash Aslam, Farhan Zaid, Ayesha Muzaffar

**Affiliations:** 1 Dr. Aliya Hisam, MBBS, MPH, FCPS. Assistant Professor, National University of Medical Sciences (NUMS), Community Medicine Department, Army Medical College, Rawalpindi, Pakistan; 2 Iqra Safoor, House Officers, Military Hospital, Rawalpindi, Pakistan; 3 Nawal Khurshid, House Officers, Military Hospital, Rawalpindi, Pakistan; 4 Aakash Aslam, House Officers, Military Hospital, Rawalpindi, Pakistan; 5 Farhan Zaid, House Officers, Military Hospital, Rawalpindi, Pakistan; 6 Ayesha Muzaffar, House Officers, Military Hospital, Rawalpindi, Pakistan

**Keywords:** Activities, Adult, Internet, Psychological, Politics, Stress, Social media, Social networking, Twitter messaging, Facebook, Social activities

## Abstract

**Objective:**

To find out the association of psychological stress with political activism on social networking sites (SNS) in adults. To find association of psychological stress and political activism with age, gender and occupational status.

**Methods:**

A descriptive cross-sectional study of 8 months (Aug 2014 to March 2015) was conducted on young adults between age group of 20-40 years of different universities of Rawalpindi, Pakistan. Closed ended standardized questionnaires (i.e. Cohen Perceived Stress-10) were distributed via non-probability convenient sampling among a total sample size of 237. Sample size was calculated using WHO sample size calculator and data was analyzed in STATA version 12.

**Results:**

The mean age of participants was 21.06±1.425 years. Out of the 237 participants, 150 (63.3%) were males and 87 (36.7%) females. Regarding their occupation, 13 (51.9%) were military cadets, 8 (3.4%) were consultant, 47 (19.8%) medical officer, 3 (1.3%) PG students and 56 (23.6%) MBBS students. Significant association of occupation was established with both political activism and psychological stress (p=0.4 and p=0.002 respectively). Among 237 individuals, 91 (38.4%) were stressed out and 146 (61.6%) were not. Among whole sample, political activists on SNS were found to be 23 (9.7%). Out of these 23 individuals who were politically active, 15 (65.2%) were stressed out and 8 (34.7%) were not. A significant association between stress and political activism was established (p=0.005).

**Conclusion:**

Political activism via social networking sites is playing significant role on adult person’s mental health in terms of stress among different occupation.

## INTRODUCTION

Stress is a state of psychological strain /tension that results from adverse demanding circumstances. Social stresses are linked to drug abuse.^1^ Social networks are a new source of psychological stress and enhancing self-esteem.^2^ The internet and in particular social networks are although an important part of daily life for both adolescents and adults but at the same time they are creating havoc for human race. Increasing use of social networking for communication among millions of people is observed. Facebook, Twitter, LinkedIn blogs are used in increasing frequency to share news and commentary.^3^ Higher levels of political beliefs indulges one’s actions and greatly augment the probability that an individual will participate in political affairs.^4^ Amon all twitter is a tool for social networking extensively.^5^ several studies have concluded that individuals experience fatigue and restlessness while frequently using these hedonic sites and can become a symbol of stress despite its voluntary usage because there is a growing disclosure of individual’s information and interests on face book, twitter etc.^3^-^9^

Len hart and colleagues explored different types of internet media practice among teenagers and young adults in United States in 2006-2010. During this time social networking sites experienced the biggest rise (an average of 50%) and the key shift in the use came at age 30 years with almost double the number of teens and 18-25 years old used them as those 30 years and over. In United Nations sixty one percent of adults search online and thirty nine percent use social media such as face book for health information.^7^ In another study, social media were used for health promotion (52%) and tools were largely community based.^8^

Access to internet is increasingly easy due to use of smart phones. In people Republic of China, the number of 3G smart phone users increased from 8% in 2008 to 95% in 2012. Amongst 55% to 82% of teenagers and young adults use Social Networking Sites (SNSs) on regular basis for example Facebook, Twitter etc. Respondents reported stress with the use of SNSs through scores ranging from 20-100. Based on Wang et al study, it was estimated that normal users had score (20-49) and problematic users had scores (above 50). Only 1% of participants (n=3) did not visited SNSs in previous three months, about two fifth (n=107) spent less than one hour/day, while 37% (n=101) spent one or two hours. 23% of participants were spending three hours/more (n=64). Twelve percent (n=33) scored 50 or above on addictive tendency scale and were classified as stressed out user also having other problems. It was concluded that SNSs contributed to three psychological risk factors, two cognitive and one personality attribute that heightens vulnerability to stress and related problem in SNSs users.^9^ Exposure to ethnic-political conflict/violence was related both to aggression and PTS symptoms, even after controlling for a range of demographic and contextual factors.^10^ Compared to non-sectarian community violence, politically-motivated community violence had distinctive influences through various mechanisms on child adjustment problems and pro social behaviour.^11^ Violence exposure in the social environment is a serious risk factor for the psychopathology development in children and adolescents.^12^ Experiences of political hardships do not increase psychosocial problems if children have strong ideological commitment.^13^ Violence stemming from ethnic and political tensions is a problem of increasing proportions throughout the world.^14^

The aim of our study was to explore what impact Social Networking Sites are producing on person’s mentality in terms of psychological stress either in a positive or negative manner among young adults (20-40 years). It is to identify how SNSs use is progressively being increased to participate in political activities as is very common in our country Pakistan like supporting and following any political party, updating statuses, commenting and in return waiting for likes, praise. Similarly activities against another party leading to anger, rage and violent reactions all culminating into stress.

## METHODS

A descriptive cross sectional study was conducted at Army Medical College Rawalpindi and Margalla College Islamabad. It was 7 months duration from August 2014 till February 2015. A sample of 225 was calculated by using WHO sample size calculator, with Confidence Level (CL) of 95%, anticipated population proportion (P) of 0.3 and relative Precision (d) of 0.2. Inflating the sample size by 10% for incomplete responses, sample size was calculated as 237. By using convenient sampling young adults of 20-40 years age group of both genders were inducted. Permission from the Ethical Review Committee of Army Medical College was taken. Verbal informed consent was taken from the administration of the institute and also from the participants. Those adults were excluded who were not using internet or did not had access to internet facility. Unwilling adults were also excluded from the study. Data collection Tool was a close ended questionnaire. Occupation was asked and participants were grouped into five categories i.e. military cadets, consultants, medical officers, postgraduate students (PG) and MBBS students. Those were identified as political activism who were using social networking sites (Facebook, Twitter, MySpace etc.) for political activities. Stress was evaluated through a standardized questionnaire that is Cohen Perceived Stress (PSS)-10 scoring.^15^ Each item of the questionnaire is rated on a five point scale; ranging from never (0) to almost always (4). Positive worded items are reverse scored, and the ratings are added, with higher scores indicating more perceived stress. PSS-10 scores are obtained by reversing the scores on the four positive items e.g. 0=4, 1=3, 2=2, etc. and then adding across all 10 items. Items 4, 5, 7, and 8 are the positively stated items. Participants having scores between 13 and 20 were categorised as not stressed out while scores more than 20 points were categorised as psychologically stressed out.

Data were entered and analysed in STATA version 12. Descriptive statistics was used to calculate mean and standard deviation of age. Frequency and percentage were calculated for qualitative variables like gender, occupation, political activists and stress. Independent sample *t* test was applied to find association of age with stress and political activity. Pearson’s chi square test was applied to find out association of gender with stress and political activism. Fisher exact test was applied for finding association of occupation with stress and political activism. A p-value of < 0.05 was considered statistically significant.

## RESULTS

The mean age of the participants was 21.06±1.425 years. Out of the 237 participants, 150 (63.3%) were males and 87 (36.7%) were females.

Regarding participants occupation, 123 (51.9%) military cadets, 8 (3.4%) were consultants, 47 (19.8%) medical officers, 3 (1.3%) postgraduate (PG) students and 56 (23.6%) were MBBS students. There were 4 (1.69%) consultant, 51 (21.52%) military cadets, 24 (10.13%) medical officer, 2 PG students and 10 (4.22%) MBBS students who were stressed out. There was a significant association between stress and occupation (p=0.002). The frequency of political activists among military cadets was 13 (5.49%) and medical officers was 9 (3.80%) while it found to be zero among consultant, PG students and MBBS students. The association between political activist and occupation was found to be significant (p=0.04).

In the whole sample, political activists were found to be 23 (9.7%) and 214 (90.3%) were not politically active. Fourteen (5.91%) male gender were politically active while 136 (57.38%) were not. About 9 (3.80%) females were politically active while 78 (32.91%) were not. The association was not significant among political activity and gender (p=0.8). There was also no significant association between age and political activity (p=0.1499).

Out of the total participants, 91 (38.4%) were psychologically stressed out and 146 (61.6%) were not. Psychological stress in males was seen in 58 (24.47%) and 92 (38.82%) were free from stress. While in females only 33(13.92%) were stressed while 54 (22.78%) were not. The association between gender and stress was not significant (p=0.911). There was also no significant association between age and stress (p=0.2375).

Out of 23 individuals who were politically active, 15 (65.2%) were stressed out while 8 (34.7%) were not stressed out. Those who were not politically active had 76 (32.07%) stressed out participants and 138 (58.23) not stressed out. A significant association between stress and political activism was established (p= 0.005).

## DISCUSSION

Our study revealed a significant rise in psychological stress in politically active SNS users. Depression is one of a devastating condition that undesirably affects different aspects of an individuals life and health. Previous researched supports the idea that there may be a relationship between the use of certain media and depression. Kramer AD et al have shown experimental evidence of massive-scale emotional contagion through social networks. Data from a large real-world social network, collected over a 20-y period suggests that longer-lasting moods (e.g., depression, happiness) can be transferred through networks although the results are controversial. Emotions expressed by people on Facebook influence others emotions, constituting experimental evidence for massive-scale contagion via social networks. In contrast to prevailing assumptions, in-person interaction and nonverbal cues are not strictly necessary for emotional contagion, and that the observation of others’ positive experiences constitutes a positive experience for people.^16^ During the last decade, there has been an immense change in the way people communicate with each other and expresses themselves through SNS Sites. SNS’s is liked to internet addiction which in turn have been linked depression, anxiety, frustration, anger, low self-esteem and many other depression symptoms in many studies but few studies contradict these findings. In our study, we have found a possible link of political activism through SNS’s with stress.^17^

**Table-I:** Association of psychological stress and political activism with demographic variables (n=237).

*Demographic Variables*	*Political activists n (%)*	*Not political activists n (%)*	*p-value*	*Stressed Out n (%)*	*Not stressed out n (%)*	*p-value*	*Total n (%)*
Age (mean ± sd)	21.06 ±1.425		0.1499*			0.2375*	
*Gender*							
Male	14 (5.91)	136 (57.38)	0.8**	58 (24.47)	92(38.82)	0.911**	150(63.3)
Female	9 (3.80)	78 (32.91)		33 (13.92)	54(22.78)		87(36.7)
Occupation							
Military cadets	13 (5.49)	110 (46.41)	0.040***	51 (21.52)	72(30.38)	0.002***	123(51.9)
Consultant	0	8 (3.38)		4 (1.69)	4 (1.69)		8(3.4)
Medical officer	0	38 (16.03)		24 (10.13)	23(9.70)		47(19.8)
PG students	0	3 (1.27)		2 (0.84)	1(0.42)		3(1.3)
MBBS students	1 (0.42)	56 (23.63)		10 (4.22)	46(19.41)		56(23.6)

Total	23 (9.7)	214(90.3)	-	91 (38.4)	146(61.6)	-	237(100)

* Independent sample *t* test,

** Pearson’s Chi square test,

*** Fisher exact test.

**Fig.1:**
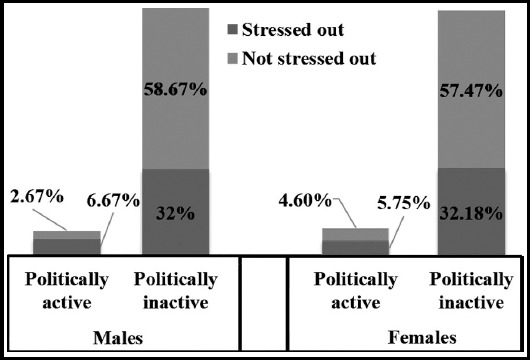
Association of psychological stress and political activisms (n=237) (p=0.005).

Park S and colleagues reported that activities on Face book reveal the depressive state of users. Face book users (male=40, female=15, mean age 24.43, SD 3.90) were recruited through advertisement fliers distributed to students in a large university in Korea. Their results using Emotion Diary app demonstrated that the more depressed one is, the more one will read tips and facts about depression. These results open the door for examining Face book activities to identify depressed individuals.^18^

Block M et al conducted a study on the relationship between self-report of depression and media usage. Data of Media Behaviour and Influence Study (MBIS) database (N = 19,776 subjects) was used and it was concluded that people who use more internet, television and social media are more prone to major depression. An association between Self-reported depression (SRD) and increased SNS was established. This study found that those who have suffered either economic or physical life setbacks in orders of magnitude more likely to be depressed, even without disproportionately high levels of media use.^19^ Our study also concluded a significant association between political activism and SNS.

Social media have both positive and negative influences on live of individuals. For those who are unable to communicate due to any reason it’s a blessing as they can communicate efficiently despite geographical distances.^20^ In our study, we did not enrolled individuals who were unable to communicate otherwise so its difficult to comment on his aspect of SNS.

People use Facebook, Twitter and other like sites to communicate, collaborate, coordinate and let their voices be heard loudly regarding their concerns. SNS have reinvented new tool for political activism. People demonstrate against their government by planning and coordinating through twitter, Facebook etc.^21^ Sriwilai K and Charoensukmongkol P. studied impacts of social media addiction on mindfulness, coping strategies and the consequence on emotional exhaustion. Results revealed that people who were highly addicted to social media tended to have lower mindfulness and tended to use emotion-focused coping to deal with stress.^22^

### Limitations of the Study

One of the study limitation was that we did not inquire regarding which SNS was used more frequently by the participants but the most active group regarding political activism were military cadets and frequency was more in males. Further studies regarding use and misuse among larger sample sizes and different groups can be conducted to find more details regarding negative as well positive aspect of political activism.

## CONCLUSION

Political activism via social networking sites is playing significant role on adult person’s mental health in terms of stress. Occupation has also significant role with stress and political activism. Social networking sites are useful in gathering information on one side but at the same time their addiction and excessive use results in increase stress levels in adults among different occupations.

## RECOMMENDATIONS

Social networking activities can be controlled and monitored. There is no problem in having political affiliation but people must adapt positive attitude, healthy discussion spending adequate time while using such sites and meanwhile should not be passing derogatory remarks against some other party, group or individual and promoting freedom of speech rather than blaming and criticizing to avoid stressful circumstances.
